# When We Should Worry More: Using Cognitive Bias Modification to Drive Adaptive Health Behaviour

**DOI:** 10.1371/journal.pone.0085092

**Published:** 2014-01-08

**Authors:** Lies Notebaert, Jessica Chrystal, Patrick J. F. Clarke, Emily A. Holmes, Colin MacLeod

**Affiliations:** 1 School of Psychology, University of Western Australia, Crawley, Western Australia, Australia; 2 MRC Cognition and Brain Sciences Unit, University of Cambridge, Cambridge, United Kingdom; University of St Andrews, United Kingdom

## Abstract

A lack of behavioural engagement in health promotion or disease prevention is a problem across many health domains. In these cases where people face a genuine danger, a reduced focus on threat and low levels of anxiety or worry are maladaptive in terms of promoting protection or prevention behaviour. Therefore, it is possible that increasing the processing of threat will increase worry and thereby enhance engagement in adaptive behaviour. Laboratory studies have shown that cognitive bias modification (CBM) can increase or decrease anxiety and worry when increased versus decreased processing of threat is encouraged. In the current study, CBM for interpretation (CBM-I) is used to target engagement in sun protection behaviour. The goal was to investigate whether inducing a negative rather than a positive interpretation bias for physical threat information can enhance worry elicited when viewing a health campaign video (warning against melanoma skin cancer), and consequently lead to more adaptive behaviour (sun protection). Participants were successfully trained to either adopt a positive or negative interpretation bias using physical threat scenarios. However, contrary to expectations results showed that participants in the positive training condition reported higher levels of worry elicited by the melanoma video than participants in the negative training condition. Video elicited worry was, however, positively correlated with a measure of engagement in sun protection behaviour, suggesting that higher levels of worry do promote adaptive behaviour. These findings imply that more research is needed to determine under which conditions increased versus decreased processing of threat can drive adaptive worry. Various potential explanations for the current findings and suggestions for future research are discussed.

## Introduction

The lack of engagement in appropriate disease prevention or health protection presents a real problem in many health domains. For example, despite melanoma skin cancer posing a serious risk, causing 48,000 deaths worldwide each year [1], many people still do not engage in simple behavioural actions that can significantly reduce the risks associated with sun exposure. Similarly, seatbelts have irrefutably been proven to save lives, yet many people still do not wear them [2]. Many health campaigns therefore endeavour to persuade people to engage in adaptive prevention or protection behaviour. Often this is done by attempting to instil a heightened sense of fear or worry by increasing people’s awareness of their susceptibility to the risk, or of the severity of the outcome. Research has shown that such ‘fear appeals’ can be quite effective in producing adaptive behaviour [3]. Indeed, the construct of worry has been identified as a driver for adaptive health behaviour. The majority of research supports a positive relationship between worry and engagement in prevention/protection behaviour, particularly in high risk populations [4].

In investigating cognitive processes underlying worry and anxiety, experimental psychopathology research has shown that these are associated with distinct processing biases [5]. Two of the most thoroughly researched of these processing biases concern attentional bias, defined as a pattern of selective attention favouring the processing of threatening information; and interpretation bias, defined as the tendency to interpret ambiguous information in a negative way. Since then, cognitive bias modification (CBM) has been established as a method for changing these dysfunctional patterns of selective information processing. CBM tasks aimed at reducing selective attention for negative information (CBM-A) repeatedly expose participants to a contingency that encourages participants to look away from threatening information and instead attend to neutral or more positive information [6]. CBM tasks targeting interpretation (CBM-I) typically encourage participants to consistently resolve emotionally ambiguous information in a more positive or benign way [7]. Laboratory studies have shown that successfully manipulating these biases to *enhance* processing of threat is associated with an increase in anxiety vulnerability, whereas modifying these biases to *reduce* processing of threat is associated with a decrease in anxiety vulnerability [6], [7]. Naturally, all clinical applications of CBM have implemented task variants designed to reduce selective processing of threat to deliver emotional benefits in clinical populations. A considerable body of literature has thus highlighted that both CBM-A and CBM-I can be used to alleviate symptoms of anxiety, depression, and excessive and pathological worry [8]. Even though studies have shown that biases can also be effectively altered to *increase* processing of threatening information, the potential benefits of this approach in encouraging adaptive behaviour have thus far not been exploited.

Cognitive models of emotion have indicated that the very same information processing biases implicated in emotional disorders can be adaptive when they target classes of information that signal legitimate danger [9]. In these cases, the absence of processing biases favouring threatening information is likely to decrease the likelihood that an individual will accurately identify a genuine threat, placing them at a greater risk of harm. As such, in these situations a “positive” bias may be maladaptive. For example, the tendency to consistently interpret instances of physical discomfort as benign may lead an individual to experience comfortably low levels of anxiety and worry, but will also prevent them from seeking appropriate medical treatment. Similarly, interpreting the change of your skin colour when out on the beach as a tan rather than as sun damage might prevent you from using appropriate sun protection. Therefore, when faced with a genuine threat, *enhanced* processing of threatening information is likely to be adaptive. The question thus arises whether CBM paradigms can be used to modify selective processing biases to target *greater* processing of information that poses a genuine threat to ultimately produce more adaptive behaviour when facing a real threat.

Given that research supports a positive relationship between worry and future prevention/protection behaviour, it is likely that the success of health campaigns will depend on the degree to which they can increase worry. Since it has been shown that a negative interpretation bias causally contributes to worry symptoms [10], [11], the aim of the current study was to investigate whether adopting a negative interpretation bias, as compared to a positive interpretation bias, can increase worry elicited by a health campaign video, and consequently increase its effectiveness in motivating adaptive behavioural patterns. To our knowledge, this is the first study to investigate the potential applied benefits of encouraging enhanced processing of threatening information via CBM.

In the current study, participants were trained to adopt either a positive or negative interpretation bias using emotionally ambiguous physical threat scenarios that remained ambiguous until the last word. An example of such a scenario reads: “You have had several minor infections recently and make an appointment to see your GP. He gives you a physical examination and makes a few notes. At the end he tells you that your physical condition is very...”. Depending on the training condition, the ambiguity in these scenarios was consistently resolved in either a benign way (i.e. “your physical condition is very good”) or threatening way (i.e. “your physical condition is very poor”) [12]. In each scenario, a few letters of the last word were omitted and participants were required to complete the word fragment in a way that was consistent with the content of the scenario. By completing 100 of these scenarios, participants are encouraged to adopt a pattern of interpretation that favours imposing either positive or negative disambiguations on emotionally ambiguous material. After this training procedure, all participants watched a melanoma campaign video provided by the cancer institute of New South Wales designed to increase perceptions of risk and severity through a graphical depiction of melanoma cells developing and spreading through the body. Worry and negative affect were assessed before and after the presentation of the video. After viewing the video, sun protection/melanoma prevention behaviour was assessed using a behavioural intentions questionnaire and a new measure of adaptive sun protection developed for the purpose of this study. This measure was designed to assess people’s prospective engagement in “sun-smart” behaviour. This second measure was developed in light of the low predictive value of traditional measures of behavioural intentions on actual behaviour [13]. In addition, many studies investigating the relationship between worry and behaviour have not explicitly differentiated the state worry and trait worry, although trait worry measures do not account for the majority of variance in state worry measures [14]. Therefore, questionnaire measures of everyday worry and trait and state anxiety were included to investigate whether state worry elicited by the video is the best predictor of behaviour, rather than general worry or anxiety.

We hypothesized that participants encouraged to adopt a negative interpretation bias regarding physical threat would experience more worry when confronted with a health campaign video than participants encouraged to adopt a positive interpretation bias. The second hypothesis was that to the degree the interpretive training and the video campaign are effective in increasing worry, people’s engagement in sun protection behaviour should be enhanced.

## Methods

### Participants

Participants were 40 undergraduate students from the University of Western Australia, participating for course credit. Participants were recruited from a pool of 831 potential candidates who were screened on trait anxiety [15] and melanoma worry (5 point scale assessing worry about melanoma skin cancer, ranging from *not at all* to *extremely*). To reduce the likelihood that participants had strong existing biases, invitations were extended to students with mid-range anxiety levels (middle third of the sample) and low to average melanoma worry (score of 1 to 3). Participants in this study were the first 40 people to accept this invitation. The final sample consisted of 9 men and 31 women, with a mean age of 18.4 (SD = 1.8). The study was approved by the ethics committee of the University of Western Australia. In accordance with the ethics requirements, written informed consent was obtained from all participants prior to the start of the study.

### Materials

**Questionnaires.** State and trait anxiety were measured using the Spielberger State-Trait Anxiety Inventory [15]. This questionnaire consists of two 20 item scales. The State scale assesses situational anxiety (asking people to report on how they feel right now) whereas the Trait scale assesses dispositional anxiety (asking people to report on how they generally feel). Items are rated on a 4 point scale resulting in scores ranging from 20 to 80, with higher scores indicating higher levels of anxiety. Both scales have good internal consistency and the STAI-trait version has demonstrated excellent test-retest reliability [16].

Worry was assessed using the Worry Domains Questionnaire short form [17]. The WDQ-SF is a 10-item measure assessing normal everyday worry including worries relating to relationships, work, future, finances and confidence. Responses are made on a 5-point scale ranging from 0 (not at all) to 4 (extremely), yielding a score of 0 – 40. The questionnaire has good psychometric properties [17], and in the current sample, Cronbach’s alpha was.91.

Mood was assessed with six items asking participants to indicate to what extent they currently experienced a particular affect. These were rated on a 9 point likert scale anchored from *not at all* to *extremely*. Three items assessed positive affect (excited, happy, enthusiastic), and three assessed negative affect (irritable, distressed, anxious). These items were delivered on four occasions across the study. For the analyses, the three items measuring positive affect were averaged to a Positive Affect (PA) score, and the three items measuring negative affect were averaged to obtain a negative Affect (NA) score. Reliability analyses showed a Cronbach’s alpha for the PA scale ranging from.873 to.893 over the 4 assessment points, while Cronbach’s alpha for the PA scale ranged from.627 to.786.

Melanoma worry was assessed using a single item asking participants to indicate how worried about melanoma skin cancer they were at present. Responses were made on a 9 point likert scale anchored from *not at all* to *extremely*.

**Interpretation bias training.** One hundred emotionally ambiguous scenarios were used to induce a positive or negative interpretation bias. The scenarios focused on physical threat and have previously been shown to be successful in inducing a differential interpretive bias [12]. Each scenario comprises three sentences which remains emotionally ambiguous until a final word that disambiguates the emotional meaning in either a threatening or benign way. An example of a scenario with the disambiguating final words in parentheses reads as follows:

“You are getting ready to go out and look at yourself in the mirror. You notice a brown mark on your face that you do not remember seeing before. It is very small and you realize it may actually be (attractive/malignant).”

Participants read each scenario, one line at a time. The last word in each scenario was presented as a fragment (e.g. “at-rac-ive” or “m-l-gnant”, depending on training condition) that participants had to complete by entering the missing letters. Participants were instructed to read the scenarios and fill in the blanks in the last word. They were informed that there was only one correct solution for each word, and that they were to use their understanding of the scenario to guide their solution of the word fragment. Following word fragment completion, a comprehension question followed which was consistent with or an extension of the disambiguated meaning. The correct answer to this question was dependent of the training direction. For example, the comprehension question associated with the above scenario is “Do you think the brown mark could be dangerous?” For participants trained to adopt a negative interpretation bias, the correct answer is “Yes”; for participants trained to adopt a positive interpretation bias, the correct answer is “No”. In each training condition, half of the comprehension questions required a yes response and half required a no response.

**Filler task.** To attenuate any potential mood differences elicited by the interpretation training, a filler task without emotional content was presented post interpretation bias training. Three digits were presented on screen and participants were required to indicate, as quickly as possible, if the majority of the digits were odd or even by pressing the left or right mouse button. Regardless of speed of response, the filler task went for five minutes and no error feedback was given.

**Melanoma video.** To assess the influence of interpretation bias training on the processing of melanoma related information designed to influence behaviour, a melanoma health campaign video was shown. This was a 30 second advisory campaign video about melanoma skin cancer, created by and provided to us by the cancer institute of New South Wales. It was created as part of the “Dark Side of Tanning” campaign released in Western Australia in the summer of 2009/2010. The aims of the campaign were to (1) Increase understanding of the severity of melanoma as a health issue, (2) Reduce pro-tanning attitudes, (3) Increase understanding of the health consequences of unsafe exposure to the sun, and (4) Increase the number of people frequently using sun protection, as well as the range of sun protection measures used. The video zooms in on a person tanning on the beach, graphically showing how cancerous cells develop and then spread through the body. The video can be viewed at the following link: http://www.youtube.com/watch?v=wJ9HkvFFgyo.

**Figure 1:**
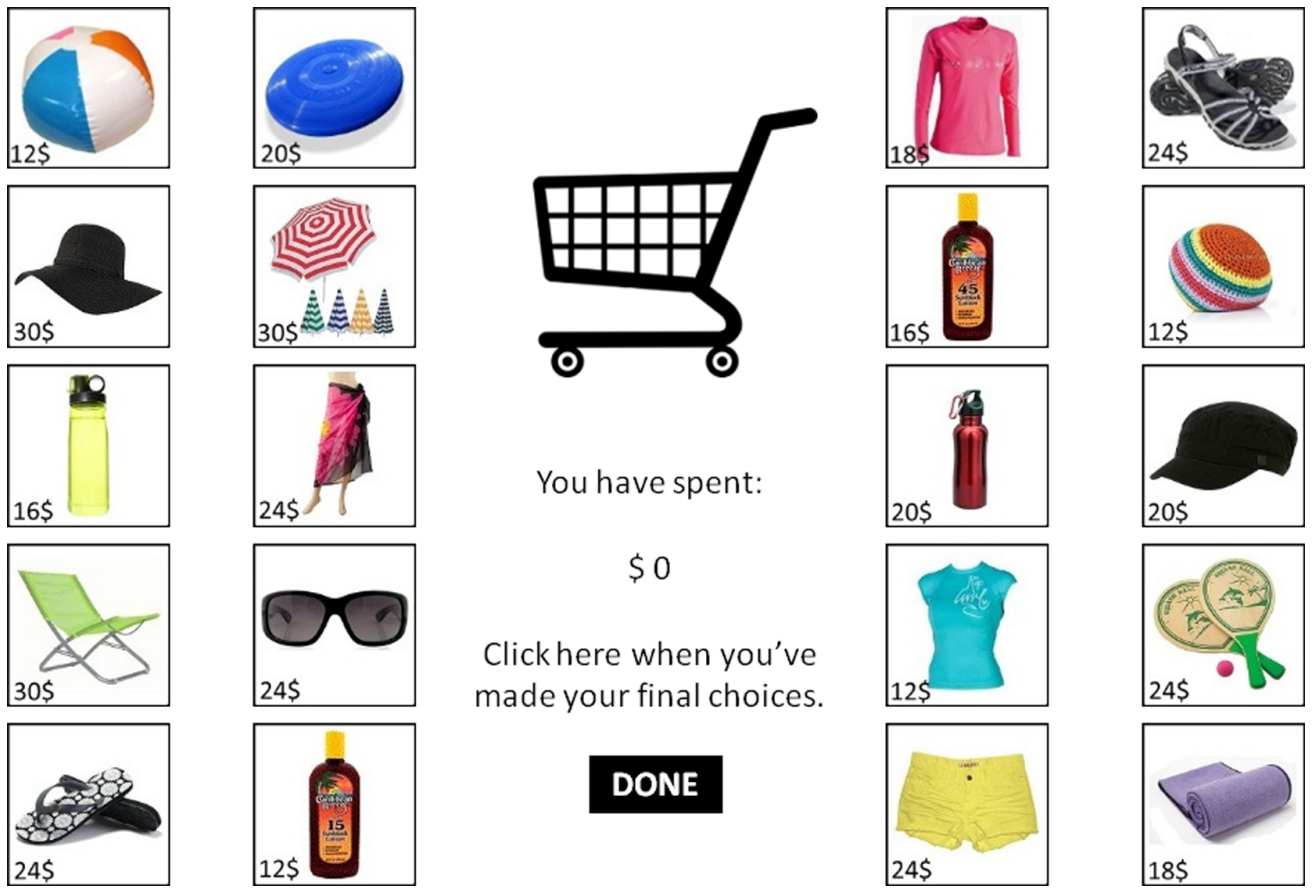
Lost luggage game.

**Behavioural assessment: Lost luggage game.** Engagement in sun protection behaviour was assessed by a game measuring preferential selection of sun protective items. The computerised task was developed for the current study. Participants were presented with a brief background story in which they were to imagine that they were going on a summer holiday in Australia and they discover upon arrival the airline had lost their luggage. As compensation the airline provided a voucher of $200AUD that could be spent on typical beach products. The items and their corresponding prices were graphically displayed on the next screen, and participants were encouraged to select items up to $200 for purchase (see Figure 1). The items included 10 sun protection measures such as sunscreen and hats; and 10 other items such as a beach ball and Frisbee. Participants were informed that each item displayed was an example item, with final colour and style to be individually determined. Each non-sun item was matched in price to a sun item. The cost of all items totalled $400 meaning that participants could only purchase a subset of items. A running total of the current amount spent was displayed, and items could be de-selected if necessary. The ratio of money participants spent on sun protection items, relative to non-sun items, served as a measure of engagement in protective behaviour.

**Behavioural intentions questionnaire.** This questionnaire consisted of five questions, gauging to what extent participants intended to engage in 5 different sun protection behaviours when exposed to harmful sunshine in the following summer. These behaviours were based on the five targets of the Australian Cancer Council, who want to encourage people to ‘*slip, slop, slap, seek, slide*’: slip on sun-protective clothing, slop on sunscreen, slap on a hat, seek shade, and slide on wrap-around sunglasses [18]. Responses were to be made on a five point scale, ranging from *Never* to *Always*. Cronbach’s alpha for these five items was.57. Item-total correlations revealed the lowest correlation for the “slop” item (r = .09). Without this item, Cronbach’s alpha increased to.64. For the analyses, all five items were included.

### Procedure

Participants were randomly assigned to a positive or negative interpretation bias training group. Participants first completed the Trait Anxiety, State Anxiety, and Worry Domains questionnaire. This was followed by the first baseline mood and melanoma worry assessment.

The interpretation bias training started with four practice scenarios in which the emotional ambiguity was resolved consistent with a participant’s training direction. The presentation of each scenario occurred in three phases. First, the initial two sentences were presented on screen for four seconds. Next, the third sentence was added without the final word for three seconds. Then, the final word fragment was added and remained on screen until response. Participants were encouraged to enter the missing letters as fast as possible and press enter. A blank screen was then presented for 500ms after which the comprehension question was presented until response. The screen display indicated a yes response required a left mouse button press, while a no response required a right mouse button press. Feedback on the accuracy of the response to the comprehension questions was presented for 1500ms. The inter trial interval was 1000ms.

The interpretation bias training consisted of two blocks with a self-paced pause in between. In the first block, 50 scenarios were presented in random order. In the second block, another 50 training scenarios were presented, randomly interspersed with 16 ambiguous test items to test whether the modification of interpretation bias was effective [7]. These test items were identical in the positive and negative training group. Half of the test items were resolved positively, and half were resolved negatively. A successful induction of a negative interpretation bias would lead to participants in the negative training group to respond faster to the negatively resolved test items than to the positively resolved test items, and vice versa for people in the positive training group. An interpretation bias index can be calculated by subtracting reaction times on positive test items from reaction times on negative test items. A higher score is thus indicative of a more positive interpretation bias. The interpretation bias training lasted approximately 30 minutes.

To assess whether negative versus positive training had a differential effect on mood and worry, a second mood and worry assessment was delivered immediately post training. This was followed by the 5 minute filler task and a third mood and worry assessment. Next, participants watched the 30 second melanoma advisory campaign video and completed the final mood and worry assessment. Participants then completed the lost luggage game and the behavioural intentions questionnaire. At the end of the session, participants were provided with the website of the cancer council of Western Australia, and the sun-smart campaign website for more information regarding melanoma skin cancer.

## Results

### Data preparation

The data of one participant was removed because they failed to enter any responses on the test items of the interpretation bias training task. For the analysis on the interpretation bias test items, extreme reaction time outliers were removed prior to analysis. Extreme outliers are defined as greater than or equal to 3 interquartile ranges above the upper quartile [19]. In the current sample, the upper quartile is 3,955, while the interquartile range is 2,027.5. The cut-off for extreme outliers was therefore 10,037.5ms. Application of this cut-off removes 27 observations. After removal of these extreme outliers, the data were shown to be not normally distributed (Shapiro-Wilk  = .841, p<.001). Therefore, analyses were performed on median reaction times as these measures are more resistant to the skew of a distribution [20].

### Baseline characteristics

Independent samples *t*-test showed that participants in the positive and negative interpretation bias training groups did not differ in terms of Trait Anxiety (*t*<1), State Anxiety (*t*<1), Worry (WDQ-SF: *t*<1; or baseline mood (PA: *t*<1; NA: *t*<1; melanoma worry: *t*(37)  = 1.45, *p*>.1), see Table 1. Further, groups did not differ in age, *t*(37)  = 1.16, *p*>.2, or gender, Chi-square (1, N = 39)  = 0.09, *p*>.7.

**Table 1:** Comparisons of the two training conditions at baseline on anxiety, worry and state affect.

	Positive CBM-I training		Negative CBM-I training	
	*M*	***SD***	***M***	***SD***
STAI-State	36.9	8.5	36.2	10.7
STAI-Trait	44.2	6.3	43.8	7.8
WDQ-SF	27.0	7.8	26.4	9.1
PA	5.1	1.8	5.5	1.5
NA	3.5	2.2	3.1	0.9
Melanoma worry	2.2	1.7	3.1	2.2

*Note* **:** STAI-State  =  Spielberger Anxiety Inventory – State version, STAI-Trait  =  Spielberger Anxiety Inventory – Trait version, WDQ-SF  =  Worry Domains Questionnaire - Short Form, PA  =  Positive Affect, NA  =  Negative Affect, Melanoma worry  =  baseline melanoma worry.

### Interpretation bias training

To assess whether an interpretation bias for physical threat had been successfully induced, a Univariate ANOVA was conducted with the interpretation bias index as dependent variable and Group (positive vs. negative training) as factor. As foreshadowed in the Method section, a larger interpretation bias index reflects faster reactions to positive than to negative test probes, and thus a more positive interpretation bias. Results showed a significant effect of Group, *F*(1,37)  = 3.97, *p* = .05, η_p_^2^ = .10, such that, as expected, the group that was trained to adopt a positive interpretation bias showed a larger interpretation bias index (*M* = 384, *SD* = 670) than the group that was trained to adopt a negative interpretation bias (*M* = −115, *SD* = 886).

A similar interpretation bias index was calculated for accuracy rates (in % correct) for the answers to the comprehension questions of the test items, by subtracting accuracy scores on negative test probes from accuracy scores on positive test scores. A larger index thus reflects more accurate responses to positive than to negative test items. A Univariate ANOVA showed a main effect of Group, *F*(1,37)  = 5.36, *p*<.05, η_p_^2^ = .13, indicating a larger index for the group that was trained to adopt a positive interpretation bias (*M* = 5.1, *SD* = 12.3) than for the group that was trained to adopt a negative interpretation bias (*M* = −3.8, *SD* = 11.6). There was no significant difference between groups in the accuracy of the response to the comprehension questions of the training items (*t*<1).

### Effects of interpretation bias training on mood and worry

A one-way analysis of covariance (ANCOVA) was performed on post-training melanoma worry scores, with training group (positive training vs. negative training) as independent variable, and pre-training melanoma worry scores entered as a covariate. There was a significant effect of training on post-training melanoma worry scores after controlling for pre-training melanoma worry scores, *F*(1, 36)  = 6.16, *p*<.05, η_p_^2^ = .15, indicating that participants in the negative training group experienced higher levels of melanoma worry after training (*M* = 2.8, *SD* = 1.6) than participants in the positive training group, (*M* = 1.5, *SD* = 1.6).

An equivalent ANCOVA on post-training NA scores revealed a significant effect of training group on NA scores after controlling for the pre-training NA scores, *F*(1, 36)  = 4.51, *p*<.05, η_p_^2^ = .11, indicating higher levels of NA in the negative training group (*M* = 3.9, *SD* = 1.3) than in the positive training group, (*M* = 3.0, *SD* = 1.3). An equivalent analysis on PA scores revealed no significant effect of training group on post-training PA scores after controlling for pre-training PA scores, *F*<1.

The filler task was effective in removing the mood effects elicited by interpretation bias training, as after the filler task participants in the two training groups did not differ in PA (*t*<1), NA (*t*<1), or melanoma worry, *t*(37)  = 1.99, *p*>.05.

### Effect of interpretation bias training on video experience

To assess the impact of the video on mood in the two interpretation bias training groups, a one-way analysis of covariance (ANCOVA) was performed on post-video melanoma worry scores, PA scores, and NA scores separately, with training group (positive training vs. negative training) as independent variable, and pre-video scores entered as the covariate.

There was a significant effect of training on post-video melanoma worry scores after controlling for the pre-video melanoma worry scores, *F*(1, 36)  = 5.88, *p*<.05, η_p_^2^ = .14. Contrary to our expectations, the results showed higher levels of melanoma worry in the positive training group (*M* = 4.7, *SD* = 1.8) than in the negative training group, (*M* = 3.3, *SD* = 1.8). An equivalent analysis on NA and PA scores revealed no significant effects of training group on post-video scores after controlling for pre-video scores, *F*s<1.

### Worry and sun protection behaviour

In the lost luggage game, the proportion of money spent on sun protection items (relative to total amount spent) served as a measure of engagement in sun protection behaviour. The average proportion of money spent on sun protection items was.59 (*SD* = .15, range.33 to.85), corresponding to an average of 109AUD (*SD* = 33, range 42 to 174). The average compiled score of reported intentions to engage in sun protection behaviour was 17.16 (*SD* = 3.05, range 10 to 25). The correlation between the two behavioural measures was not statistically significant, *r*(31)  = .34, *p* = .06 (due to technical difficulties, data from seven participants on the behavioural intentions measure were lost). For both measures, there were no significant differences between training groups, *t*s<1.

To assess which cognitive or emotional constructs might be associated with adaptive sun protection, correlational analyses were performed with the two sun protection measures: the proportion of money spent on sun items in the lost luggage game (calculated as money spent on sun items divided by the total amount of money spent), and the total score of the five behavioural intentions questions. The correlation between these sun protection measures and anxiety measures, worry measures, worry and NA elicited by the melanoma video (measured as pre video scores subtracted from post video scores), and the reaction time index of interpretation bias was investigated. For an overview of these results, see Table 2. The strongest correlation was observed between video-elicited melanoma worry and the proportion money spent on sun protection in the lost luggage game, *t*(39)  = .42, *p*<.01, indicating that the more participants increased in melanoma worry because of the video, the more they spent on sun protection in the game afterwards.

**Table 2:** Correlation between the two measures of engagement in sun protection with the anxiety, mood, worry and interpretative bias measures.

	Proportion sun expenditure	Behavioural intentions∧
STAI-Trait	−.15	.02
STAI-State	−.13	.09
WDQ-SF	.12	−.34^+^
Interpretation bias index (RT)	−.03	−.23
Video elicited Melanoma worry	.42**	.23
Video elicited NA	.26	−.17

*p*<.01, ^+^.05<*p*<.1.

∧ The same pattern of results was observed when excluding the “Slop” item from the behavioural intentions measure.

## Discussion

### The relationship between interpretation bias and worry

The aim of the current study was to investigate whether training people to adopt a negative as opposed to a positive interpretation bias can increase the effectiveness of a health campaign video by inducing more worry. We hypothesized that (1) participants encouraged to adopt a negative interpretation bias would experience more worry when confronted with a health campaign video than participants encouraged to adopt a positive interpretation bias and (2) that the more worry was elicited by the video, the more participants would engage in adaptive behaviour. Results showed that contrary to our first hypothesis, participants in the positive training group reported higher levels of worry in response to the melanoma video than participants in the negative training group. Regarding the second prediction, a bigger increase in worry was indeed associated with more engagement in sun protection as measured by the lost luggage game.

Surprisingly, the pattern of findings with regards to the first hypothesis was opposite our predictions. Previous research has shown that encouraging people who suffer from pathological levels of worry to adopt a positive interpretation bias leads to fewer worry symptoms [10], [11]. In contrast, the current study found that the induction of a positive interpretation bias led to a bigger increase in worry when watching a health campaign video than adopting a negative interpretation bias. Several explanations for this effect can be considered. First, previous studies that have influenced worry through positive CBM-I training have focused on reducing maladaptive, pathological worry in clinical or sub-clinical samples [10], [11]. In these studies, worry was operationalized as the number of negative thought intrusions experienced during a breathing focus task. This type of intrusive worry is considered maladaptive as it is irrelevant to the present task and goals. However, it is possible that the type of worry induced through health campaigns is different from such maladaptive worry in that it is solution-focused in relation to a specific and real threat. As such it is possible that this type of adaptive worry might be triggered by different mechanisms. This may have been the case in the current study where the type of worry assessed was very relevant given the context (the melanoma video). It has been suggested that rumination, a term often used to define worry, can be divided into three categories. Action rumination is focused on correcting past mistakes and achieving current goals, state rumination focuses on the implications of failure, and task-irrelevant rumination can serve to distract from a failure experience and is focused on events or people unrelated to the failure experience [21]. Research has shown that action rumination can lead to performance improvement relative to the two other types of rumination when participants are given the opportunity to repeat a task after they have been given failure feedback on the first task completion [22]. Hence, when faced with a problem or threat, some types of worry might be adaptive whereas others might be maladaptive. The type of worry assessed in the Hirsch et al. [10] and Hayes et al. [11] studies might be classified as task-irrelevant rumination, which is maladaptive. Given that worry in the health domain is known to facilitate adaptive behaviour, it is possible that this type of worry is more similar to action rumination. Perhaps to increase this action rumination in non-clinical samples, different mechanisms need to be targeted as compared to when decreasing maladaptive task-irrelevant rumination. This is yet to be investigated.

Another possible explanation for lower worry produced by negative training compared to positive training, relates to the relative effectiveness of fear campaigns. It is interesting to note that fear appeals tend only to promote protective behaviour when people believe that there are actions that can be undertaken to mitigate the risk that is communicated, and when people believe they care capable of performing those actions [3]. In the absence of such belief in self-efficacy, strong fear appeals may promote behavioural avoidance rather than engagement. For example, a study examining responses to threatening health information showed that smokers exposed to threatening smoking-related pictures were better able to disengage their attention from these stimuli than non-smokers, indicating facilitated avoidance of fear-related stimuli in those for whom the threat is particularly relevant [23]. Thus, one possible account of the current results is that training through negative CBM-I increased the personal salience of negative health outcomes. Hence, when a fear campaign was presented, those exposed to the negative CBM-I (for whom the threat may be most relevant) actually avoided the message of the video as an emotion regulation strategy and were consequently less worried by its content than the positive training group. While the data cannot determine whether this interpretation is correct, it presents an important consideration in the planning of future studies. For example, a manipulation of self-efficacy might be included before the health promotion message, by conveying that adhering to the appropriate norms for sun protection is either very difficult or very easy. If low self-efficacy indeed contributes to avoidance of health threat information, the latter message may lead to less avoidance and more worry as compared to the former message (many thanks to the Reviewer Louise Sharpe for this interesting suggestion).

A third issue which could potentially bear on the present pattern of findings regarding CBM-I and worry, is the relative match between the training context and the context of the emotional experience. In a recent study, Mackintosh, Mathews, Eckstein, & Hoppitt [23] (Experiment 3) demonstrated that CBM-I only influenced emotional vulnerability to a failure experience when the content of the training and the content of the emotional experience were matched. Specifically, only when the training scenarios involved interpreting coping with failure in a benign versus negative way, was a training-congruent difference in emotional responding to the failure experience observed. Without such specific matching of the content of the interpretations targeted in the training scenarios and the stressor experience, there was no influence of training on emotional vulnerability (Experiment 1), or –similar to the current study- a reverse effect was obtained such that participants in the positive training group showed a larger increase in negative affect than participants in the negative training group (Experiment 2). This reversed effect was attributed to the contrast between the training scenarios which involved imagining examination/test successes (positive training) or failures (negative training) and the stressor task (a test failure experience). After strengthening an optimistic bias during interpretation bias training, participants in this positive training condition may have experienced greater violation of such positive expectancies by the unambiguously negative failure experience that followed, whereas participants in the negative training condition may have come to expect such aversive outcomes and therefore experienced a lesser increase in negative affect as a result of the failure experience [23]. Similarly in the current study, there was a stark contrast between the content of the training in the positive interpretation bias training condition (positive outcome to potential physical threats) and the content of the video (unambiguously negative health threat). As with the findings of Mackintosh et al. [23], it is possible that establishing a positive interpretation bias via repeated exposure to positive/benign scenarios leads to greater violation of positive expectancies in response to the threatening melanoma video. This surprise effect may then contribute to higher levels of worry elicited by the video.

### The relationship between worry and behaviour

The second hypothesis relating to the relationship between worry and adaptive behaviour was supported: there was a moderate (.42) positive correlation between melanoma worry elicited by the melanoma video and subsequent engagement in sun protective behaviour as evidenced by the proportion of funds spent on sun protection items in the lost luggage game. There was a non-significant positive correlation between elicited melanoma worry and behavioural intentions (.23). Sunsmart behaviour was not associated with state or trait anxiety, and general worry was negatively correlated with behavioural intentions. As such, domain-specific state worry proved the best predictor of adaptive behaviour.

The positive correlation between melanoma worry and engagement in sun protective behaviour is consistent with other findings that have shown that melanoma worry predicts greater engagement in sun protection behaviour including the uptake of a sunscreen coupon in sunbathers [24], and skin cancer screening clinic attendance [25]. McCaul and Mullens [26] offer several explanations for why worry might drive adaptive behaviour. Firstly, the experience of worrying over something can be an extra reason to take health protective action on top of already existing reasons, and the more reasons there are to take action, the more likely it is that action will be undertaken. Secondly, as worry involves uncontrollable repetitive intrusive thoughts about the risk or danger, worry may keep the issue salient and thus serve as an active reminder that something needs to be done. This way, worry might act as an ongoing cue to take action. Thirdly, worry might produce mental simulations of the risk or danger, including potential solutions to the problem [27]. This problem-solving component might explain why worry, as opposed to anxiety, can drive adaptive behaviour.

### Strengths, limitations, and future research

The behavioural measure developed for the current study was designed to provide an indication of future actual behaviour rather than being limited to assessing past behaviour or behavioural intentions [13]. Future research however could usefully investigate the extent to which performance on this behavioural measure corresponds to actual behaviour. A follow-up investigation of the degree to which performance in the game versus self-reports of behavioural intentions predicts actual behaviour would be a valuable test of the ecological validity of these behavioural measures. Preliminary results of a validation study do show a significant correlation between the proportion of money spent on sun protection in the game measured at the start of summer and self-reported sun protection behaviour measured at the end of summer, r(66)  = .37, p = .002. Follow-up measures of melanoma worry may also shed light on the potential long term impact of differential interpretation bias training. Perhaps the temporal proximity and the unambiguously negative nature of the video caused this surprising pattern of results, whereas in the longer term, a negative interpretation bias may still lead to greater worry and subsequent enhanced engagement in sun protective behaviour.

The sample size in the current study was rather small, although it was sufficient to detect a medium sized effect with a power level of.80 [28]. A second potential limitation of the current study is that only one measure of interpretation bias was included to assess the effectiveness of the interpretation bias manipulation. Other interpretation bias training studies have included other measures that are less sensitive to response bias effects, such as a recognition memory task. However previous research has shown that modification of interpretation bias can be observed both in responses to positive and negative probe sentences, and a recognition memory task [7]. It is possible however, that the mere exposure to positive and negative scenarios in the two training groups could lead to the observed pattern of interpretive bias. After all, perhaps simply repeatedly exposing participants to either positive or negative scenarios would be sufficient to produce a valence-congruent bias that would appear to be a differential interpretation bias. However, recent research has shown that repeated exposure to positive or negative scenarios alone is not sufficient to alter perceptions of subsequent ambiguous information and the presence of ambiguity in the training scenarios is crucial to produce a differential training effect [29]. This provides some reassurance that the pattern of induced interpretive bias observed in the current study was indeed genuine.

To our knowledge, only two studies to date have investigated the relationship between interpretation bias modification and behaviour, and both were aimed at discouraging maladaptive behavioural patterns associated with levels of worry and anxiety that are too high (avoidance in people with phobias) [30], [31] rather than discouraging maladaptive behavioural patterns associated with levels of worry and anxiety that are too low. Both studies failed to find an effect of the modification of interpretation bias on behaviour, but in one of these studies [31] it was argued that changing automatic or reflexive behaviour through CBM-I might not be effective because these behaviours could be independent of the cognitive processing of threatening information. Briefly enhancing or attenuating the processing of threatening information through CBM might therefore have little to no effects on this type of automatic behaviour and perhaps repeated training sessions are needed to achieve such change. In contrast, modifying patterns of cognition, by its very name, might be effective in targeting behavioural change when the behaviour has a cognitive basis. Indeed in the current study, the targeted behaviour is dependent on intentional, conscious decisions to engage in health protection. Similarly, mental imagery training has been shown to influence behaviour with a cognitive basis [32]. People with dysphoria who were trained to generate mental images in response to positive image-word cues showed better performance (caught more fish) in a subsequent fishing game than people in the negative training or control conditions. Performance in this fishing game reflected persistence, and is hence also cognitively based. Thus, separate to the issue of whether the stimulation of adaptive behaviour is best encouraged by enhancing or attenuating processing of threatening or positive information, such modification of cognitive processes might be selectively efficacious in changing behaviour that has a strategic, cognitive component, rather than automatic behavioural patterns.

The above mentioned study also highlights that other CBM paradigms might also be effective in targeting behavioural change in the context of health psychology. For example, attentional bias modification has been shown to increase job performance in a highly stressful workplace [33] (study 3b). If CBM-A can indeed affect self-esteem, decrease stress and thereby increase optimal performance, the question remains whether it can also affect emotional and cognitive responding in a way that would optimise patterns of behaviour in the context of health promotion or disease prevention. Future studies could thus investigate the effectiveness of other CBM paradigms to target health behaviour. Independent of the paradigm used, future studies should consider devising material that matches closely in content to the type of behaviour that is targeted as research has shown that matching the content of training to the intended target for change is more likely to be effective [23]. For example, when targeting sun protection behaviour, a content-matched training scenario could read as follows: “You feel the heat of the sun on your skin when you are at the beach in the middle of a summer day. Considering the time since you last put on sunscreen, your chances of getting burnt are high/low”.

The current study presents a first investigation into increasing worry through cognitive bias modification for interpretation to effect an increase in adaptive health protection behaviour. Although contrary to expectations a bigger increase in melanoma worry was achieved in the positive rather than the negative interpretation bias training condition, more worry was associated with more adaptive behaviour as assessed with a new prospective measure of sun protection behaviour. Future studies should investigate which cognitive bias modification paradigms are most effective at targeting health related prevention/protection behaviour, and which types of worry need to be amplified to fuel this adaptive behaviour.
